# Institutional Housekeeping

**Published:** 1912-02-17

**Authors:** 


### INSTITUTIONAL HOUSEKEEPING.

**(Workers' Questions and Criticism of every kind will be welcomed.)**

### The Burden of Adulteration.

The weight of the housekeeper's responsibilities in regard to the food supply can only be properly estimated by taking into consideration the prevalence of adulteration. In the larger institutions employing a trained steward it is probable that this danger is sufficiently guarded against. But in smaller institutions all the factors which favour the adulteration of foodstuffs are present, and the consumers fall a ready prey to unscrupulous traders.

The favouring conditions for adulteration are (*a*) low prices; (*b*) lessening of competition; (*c*) inexperience in the framing of contracts and the selection of goods; (*d*) delivery in bulk and want of watchfulness in those charged with receiving the goods; (*e*) powerlessness on the part of the actual consumer, who has no voice in the selection.

### Scientific Adulteration.

When it is remembered with what care it is necessary for the consumer to choose goods intended for his own private use, how easy it is to get an inferior article substituted in place of what has been paid for, it will be understood how greatly the hospital is hampered by the above disadvantages. The extent to which the consumer is exposed to fraud is exemplified in the recent report of the Local Government Board, whose inspectors are ceaselessly vigilant in carrying out supervision of the meat and milk supply, and the provisions of the Sale of Food and Drugs Act. In all 100,749 analyses were made of milk, lard, margarine, etc., flour, spirits, butter, beer, coffee, pepper, and drugs. Out of these samples no fewer than 8,252 were reported against, or 8.2 per cent. It is very necessary for those who are charged with the reception of large supplies to keep pace with the tricks of the trade as revealed by the inspectors. Most hospitals check the quality of their milk by frequent analyses. It is instructive to learn that those who practise adulteration are becoming very wary and even scientific in their methods. The plan adopted is to tone all the milk down to the "extreme margin of the Board of Agriculture's limits of 8.5 per cent. of non-fatty solids, and 3 per cent. of fat." This is done by the addition of separated milk, until genuine milk of a rich quality is reduced to the poorest quality permissible by law. There can be no difficulty, if proper precautions are taken, in recognising that this practice is at work.

Another common device of the trader is to sell a different article to that which has been asked for, protecting himself by making a declaration which may or may not be observed by the purchaser. Thus when coffee was asked for, a mixture of coffee and chicory was sold in 50 per cent. of the instances, and in most of them a declaration to that effect was supplied. It is a main principle of the Sale of Food and Drugs Act that a purchaser is entitled to receive the article which he has demanded, but it is easy to estimate what chance there is of detection in a busy hospital where the goods are purchased by one individual, received by a second, and used by a third.

The adulteration of butter by water is on the increase. The legal limit is 16 per cent., and precautions should be continually taken to guard against this being exceeded. Out of 1,048 samples of butter reported against in 1910, 830 contained foreign fat, either cocoa-nut oil or beef or mutton oleo and stearin. The process is so ingenious that it is said even an analyst, relying upon preliminary tests, might be led to pass such a fat as pure butter fat. The point for the housekeeper to consider is that margarine ought to be about half the price of butter, and she ought not to pay at the pure butter rate.

Cheese is an important article of diet for the use of the staff in institutions, and its manifold forms of adulteration must not be overlooked. Cheap Cheshire cheese has been found to contain a minimum of fat and the very maximum of water, "45 per cent. of the latter, and as low as 4 per cent. of the former"; whereas Cheshire cheese ought not to contain less than 20 per cent. of fat.

### Managers' Duties.

To avoid adulteration it is necessary in the first place to guard against attempting to force down the prices lower than will admit of a fair profit to the tradesman. Secondly, it is highly important, when goods are procured from local sources, to test very carefully the samples sent in instead of mechanically accepting year after year the usual contract. It is always fair to the local tradesmen to give them the order if they can work up to a sound level of quality, but in the majority of institutions very little trouble is taken about the contracts, and things drift on far below the standard warranted by the price paid. Lastly, there is too frequently grave absence of precautions in the reception of goods, whether they be daily supplies or quarterly consignments. In order to exercise a sufficient check it is indispensable that one person should be entirely responsible, and should have leisure to examine into and supervise ceaselessly the quality of all goods supplied to the institution.

How is it possible for a busy matron to overhaul systematically the various packages of groceries and other goods which await her attention in the storeroom or receiving-rooms, examine the butter, test the milk, weigh the bread? And yet if any of these precautions be omitted the hospital is exposed to fraud. It is much if the matron who has no housekeeper can manage through the agency of porter or probationer to check the actual quantities received.

These matters demand concentration, business ability, a trained eye, hours of close application. A housekeeper devoting entire time to selecting, receiving, and checking necessary articles, would more than pay her own salary.

